# Time-dependent association between STOPP and START criteria and gastrointestinal bleeding in older patients using routinely collected primary care data

**DOI:** 10.1371/journal.pone.0292161

**Published:** 2023-12-07

**Authors:** Anouk Veldhuis, Danielle Sent, Rik J. B. Loijmans, Ameen Abu-Hanna

**Affiliations:** 1 Department of Medical Informatics, Amsterdam UMC Location University of Amsterdam, Amsterdam, The Netherlands; 2 Department of General Practice, Amsterdam UMC Location University of Amsterdam, Amsterdam, The Netherlands; University of South Australia, AUSTRALIA

## Abstract

**Purpose:**

Only few studies have assessed the preventive effect of the STOPP/START criteria on adverse events. We aim to quantify 1) the association between nonadherence to STOPP/START criteria and gastrointestinal bleedings, and 2) the association between exposure to the potentially harmful START-medications and gastrointestinal bleedings.

**Design:**

A retrospective cohort study using routinely collected data of patients aged ≥ 65 years from the electronic health records (EHR) of 49 general practitioners (GPs) in 6 GP practices, from 2007 to 2014. The database is maintained in the academic research network database (AHA) of Amsterdam UMC, the Netherlands.

**Methods:**

Gastrointestinal bleedings were identified using ICPC codes and free text inspections. Three STOPP and six START criteria pertaining to gastrointestinal bleedings were selected. Cox proportional hazards regression with time-dependent covariate analysis was performed to assess the independent association between nonadherence to the STOPP/START criteria and gastrointestinal bleedings. The analysis was performed with all criteria as a composite outcome, as well as separately for the individual criteria.

**Results:**

Out of 26,576 participants, we identified 19,070 Potential Inappropriate Medications (PIM)/Potential Prescribing Omission (PPO) instances for 3,193 participants and 146 gastrointestinal bleedings in 143 participants. The hazard ratio for gastrointestinal bleedings of STOPP/STARTs, taken as composite outcome, was 5.45 (95% CI 3.62–8.21). When analysed separately, two out of nine STOPP/STARTs showed significant associations.

**Conclusion:**

This study demonstrates a significant positive association between nonadherence to the STOPP/START criteria and gastrointestinal bleeding. We emphasize the importance of adherence to the relevant criteria for gastrointestinal bleeding, which may be endorsed by decision support systems.

## 1. Introduction

Polypharmacy is an increasing problem amongst older people [1]. In the Netherlands, 10% of patients between 40 and 65 years of age use at least five different medications. This percentage shoots to 30–45% for patients aged 65 or more [2,3]. Although medication is intended to benefit the patient, adverse drug reactions (ADR) may occur [4]. The odds for older patients to be hospitalized because of ADRs is four times higher when compared to younger individuals [5]. 50–88% of the hospitalisations of older patients due to ADR are regarded as preventable [5–7]. The most common manifestation of ADR in older people are falls, orthostatic hypotension, delirium, renal failure, gastrointestinal bleedings and intracranial bleedings [5]. Of which gastrointestinal bleeding most frequently leads to hospitalization and death [5,7].

Several prescription guidelines have been designed to decrease the number of potentially harmful ADRs [8–10]. These guidelines contain criteria to prevent potentially inappropriate prescribing (PIP), in the form of potentially inappropriate medications (PIM), potential prescribing omissions (PPO), or both. One of the most commonly used prescription guidelines in Europe are the STOPP (Screening tool of older person’s prescriptions) and START (Screening tool to alert doctors to right treatment) criteria ([8,11]. There are 65 STOPP criteria pertaining to PIM, and 22 START criteria concerning PPO [11]. Since its publication, multiple studies have focussed on assessing the prevalence of the (inadherence to the) STOPP and START criteria (hereafter STOPPs and STARTs), clinical outcomes, health care utilization and quality of life [12]. Almost all of these studies use the guideline as a whole, with no distinction in outcome among different STOPPs or STARTs [12]. The most considered clinical outcomes are falls and delirium. Other ADRs like gastrointestinal bleedings are ignored [12,13]. The reported prevalence of PIMs and PPOs is estimated between 21% and 85% (CI 19% - 91%) [14–17]. The wide range in prevalance estimates in these studies can be attributed to variations in patient characteristics, healthcare settings and, most importantly, details of the criteria’s application [12,18]. Variation in the criteria’s application can be overcome by using standardized terms for medications, such as the Anatomical Therapeutic and Chemical (ATC) codes, and diagnoses, such as the International Classification of Primary Care (ICPC) [8,18]. In 2014, the STOPP and START guidelines were adapted for the Dutch situation and specified using the ATC and ICPC codes [14,18]. We use this version of the criteria in this study.

Studies focusing on the association between ADRs as clinical outcome and nonadherence to the STOPP and START guideline are sparse. Some STOPPs or STARTs might be fairly common, but their effect on the patient’s risk of ADRs like gastrointestinal bleedings, is still unknown. In our previous conference article, we addressed the association between nonadherence to STOPPs and STARTs and gastrointestinal bleeding, focussing on the larger overall effect, per year [19]. The results of that study motivated this second more complex and time dependent study that relied on additional data. The primary aim of this second study is to quantify the association between nonadherence to the respective STOPPs and STARTs (as a composite outcome) and gastrointestinal bleedings. Our second primary goal is to quantify the association between the (potentially harmful) medications of the individual STARTs and gastrointestinal bleedings. A secondary aim of this study is to quantify the association between nonadherence and gastrointestinal bleedings for the individual STOPPs and STARTs

## 2. Methods

### 2.1 Study setting and population

We performed a retrospective cohort study using routinely collected data from the academic general practitioners’ research network (AHA) of the department of General Practice at Amsterdam UMC, location AMC in Amsterdam, the Netherlands (https://www.vumc.nl/anh/database-anha.htm). This network contains anonymized electronic health records of more than 182,000 patients of 49 GPs in the southeast of Amsterdam. The dataset includes date of birth, gender, current diagnoses and medical history as coded according to the ICPC, consultation notes, results of diagnostic tests and drug prescriptions. The drug prescription records include ATC codes, dosage, and prescription start and end dates. To be able to build upon our earlier preliminary work [19], we used the same dataset we used before. All patient records that dated from January 1^st^ 2007 up until December 31^st^ 2014 were used. All patients aged ≥ 65 years were included, either at the start of the study, or from the moment they turned 65 years of age within the period of the study. Participants were followed until the study end date, death, or leaving the participating GP practice, whichever occurred first.

### 2.2 Criteria and diagnosis selection

We selected three STOPPs and six STARTs that aim to decrease the risk of gastrointestinal bleeding. Table 1 describes the criteria and the respective codes used in the guideline. These STOPPs and STARTs were translated into computer scripts with the prespecified ATC and ICPC codes in the adapted Dutch version of the criteria as described in earlier studies [8,14,18]. For each PIM or PPO, the start and end date of medication prescriptions, participant number and the corresponding STOPP or START code were obtained. The occurrence of gastrointestinal bleeding was defined as hematemesis (ICPC-1 D14), melena (ICPC-1 D15), duodenal ulcer (ICPC-1 D85) or other stomach ulcers (ICPC-1 D86). To cope with potential mismatches between diagnosis and expected ICPC, we searched the free text of the GP’s journals for keyword combinations of the Dutch translation of “bleed” and “stomach”, “digest”, “gastro” or common abbreviations of “tract” and “digest” [20]. Each resulting combination of keywords and a general, non-specific ICPC code was included, excluding the combinations that contained a specific ICPC code for a different diagnosis. For each gastrointestinal bleeding, a unique event number, the event date and the participant number were registered.

**Table 1:** The selected PIM and PPO criteria with their respective STOPP and START codes used in the STOPP and START guideline.

**STOPP codes**	**Description of PIM criterion**
STOPP A9	ASA and dose >160mg/day or ECC dose > 200mg/day
STOPP A15	platelet aggregation inhibitors or oral anticoagulants and concurrent bleeding disorder
STOPP E5	NSAID during >3 months for gout without contraindication or proven ineffectiveness for allopurinol
**START codes**	**Description of PPO criterion**
START D2A	NSAID and history of peptic ulcer disease or complication from peptic ulcer disease without PPI
START D2B	patients aged >70 years using NSAID without PPI
START D2C	NSAID in patients 60–70 years and oral anticoagulants or oral corticosteroids or SSRI or ASA or ECC without PPI
START D3A	ASA or ECC and >60 years and history of peptic ulcer without PPI
START D3B	ASA or ECC and >70 years and oral anticoagulants or oral corticosteroids or SSRI without PPI
START D3C	ASA or ECC and >80 years without PPI

Medications are abbreviated as follows: Acetylsalicylic acid–ASA, effervescent calcium carbasalate–ECC, Non-steroid anti-inflammatory drugs—NSAIDs, Proton pump inhibitor–PPI, and Selective serotonin reuptake inhibitor—SSRI.

**Fig 1:**
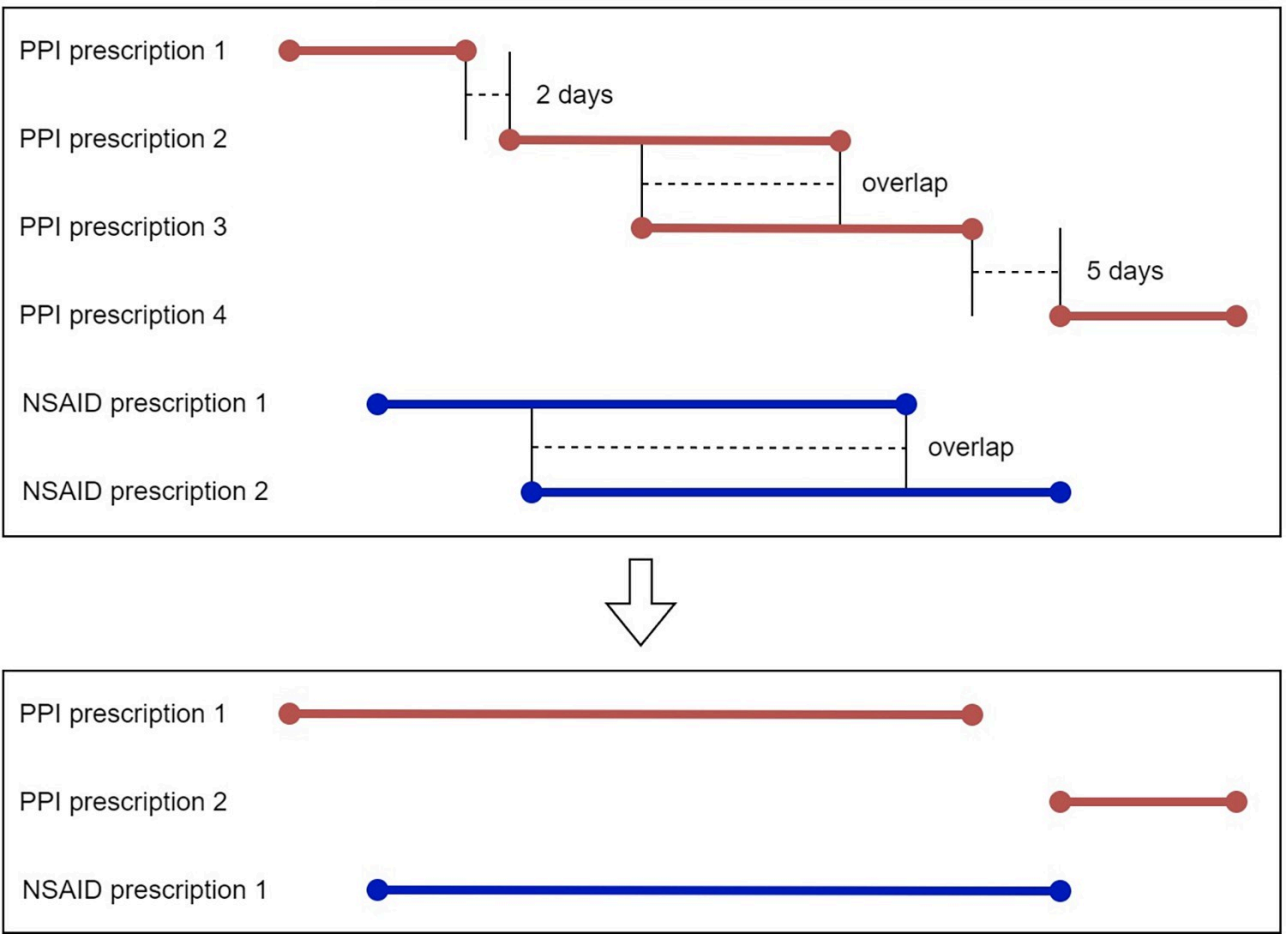
Merging process of overlapping or adjacent prescriptions for the same medications in the same patient. In cases where the end date of the first prescription and the start date of the second prescription were less than (at most) five days apart (depending on the medication), prescriptions were merged, concerning the same medication type and the same patient. We assumed this would concern chronic medications, used uninterruptedly by the patient, for which a new prescription was issued by the GP. The number of days forming the “gap” was chosen based on the clearance properties of each medication. The number of days chosen for each medication can be found in Table 2. For PPI, the maximum number of days between prescriptions for merging is 3. Medications are abbreviated as follows: Non-steroid anti-inflammatory drugs–NSAIDs and Proton pump inhibitor–PPI.

**Fig 2:**
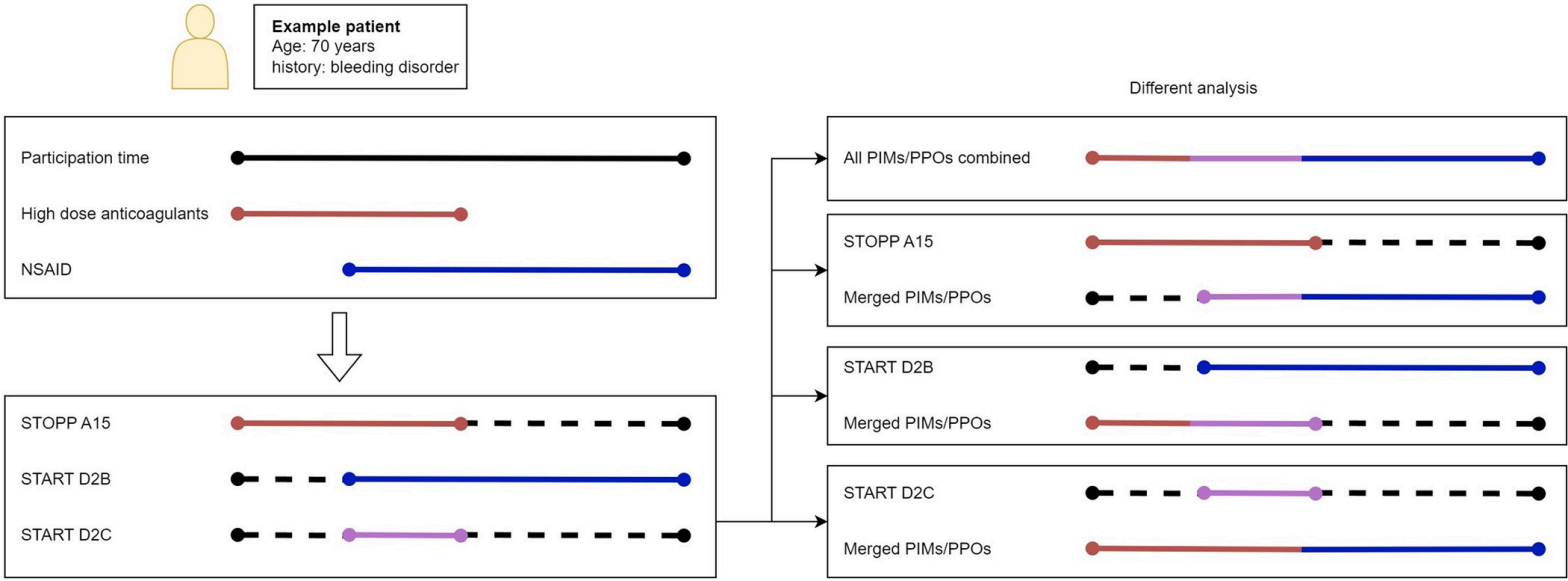
Example of merging different PIMs and PPOs together to use as confounder during analysis of separate STOPP or START criteria, as well as merging all overlapping PIMs and PPOs for analysis as an ensemble.

Each of the medication prescriptions contains dispensing instructions, number of pills, a start date and an end date. Overlapping or (near) consecutive prescriptions were merged, as seen in Fig 1 and Table 2. For analysis of all STOPPs and STARTs combined, we merged overlapping STOPPs and STARTs for each participant, using the earliest start date and latest end date. Fig 2 shows an example of merging overlapping PIMs or PPOs: a 70-year-old patient with a concurrent bleeding disorder might have had a prescription for high dose anticoagulants (STOPP A15) in the first two weeks of January and a prescription for an NSAID without a PPI (STARTD2B and START D2C) for the last three weeks of January. The patient would have three PIM or PPO records: STOPP A15 for the first two weeks of January (high dose anticoagulants), START D2B for the last three weeks of January (NSAID without PPI in patients over 70) and START D2C the second week of January (combining anticoagulants and NSAID without PPI). For the analysis of all PIMs and PPOs as an ensemble this would result in one record of a PIP for the whole of January for this patient. When the separate PIMs or PPOs are considered, all other PIMs and PPOs for that patient were merged and considered as a confounder. In our example, when analysing STOPP A15, the records for START D2B and START D2C would be merged into one record of a PIP for the last three weeks of January. All overlapping PIMs or PPOs of the same type occurring with the same participant were merged in advance using the first start date and last end date. Abbreviation: Non-steroid anti-inflammatory drugs–NSAIDs.

**Table 2:** The days added to the expiration date of the prescription per medication per STOPP or START criterion.

PIM or PPO code	PIM or PPO criterion	Medication	Days added to end date
STOPP A9	ASA and dose >160mg/day or ECC dose > 200mg/day	ASA, ECC	5
STOPP A15	platelet aggregation inhibitors or oral anticoagulants and concurrent bleeding disorder	Platelet aggregation inhibitor	4
STOPP E5	NSAID during >3 months for gout without contraindication or proven ineffectiveness for allopurinol	NSAID (use: > 3 months, chronic)	3
START D2A	NSAID and history of peptic ulcer disease or complication from peptic ulcer disease without PPI	NSAID	3
		PPI	3
START D2B	patients aged >70 years using NSAID without PPI	NSAID	3
		PPI	3
START D2C	NSAID in patients 60–70 years and oral anticoagulants or oral corticosteroids or SSRI or ASA or ECC without PPI	NSAID	3
		Oral anticoagulants, corticosteroids, SSRI, platelet aggregation inhibitor	2
		PPI	3
START D3A	ASA or ECC and >60 years and history of peptic ulcer without PPI	ASA, ECC	5
		PPI	3
START D3B	ASA or ECC and >70 years and oral anticoagulants or oral corticosteroids or SSRI without PPI	ASA, ECC	5
		Oral anticoagulants, oral corticosteroids, SSRI	2
		PPI	3
START D3C	ASA or ECC and >80 years without PPI	ASA, ECC	5
		PPI	3

Medications are abbreviated as follows: Acetylsalicylic acid–ASA, Effervescent carbasalate calcium–ECC, Non-Steroidal Anti-Inflammatory Drug–NSAID, Proton Pump Inhibitor–PPI, and Selective Serotonin Reuptake Inhibitors—SSRI.

### 2.3 Data analysis

**Fig 3:**
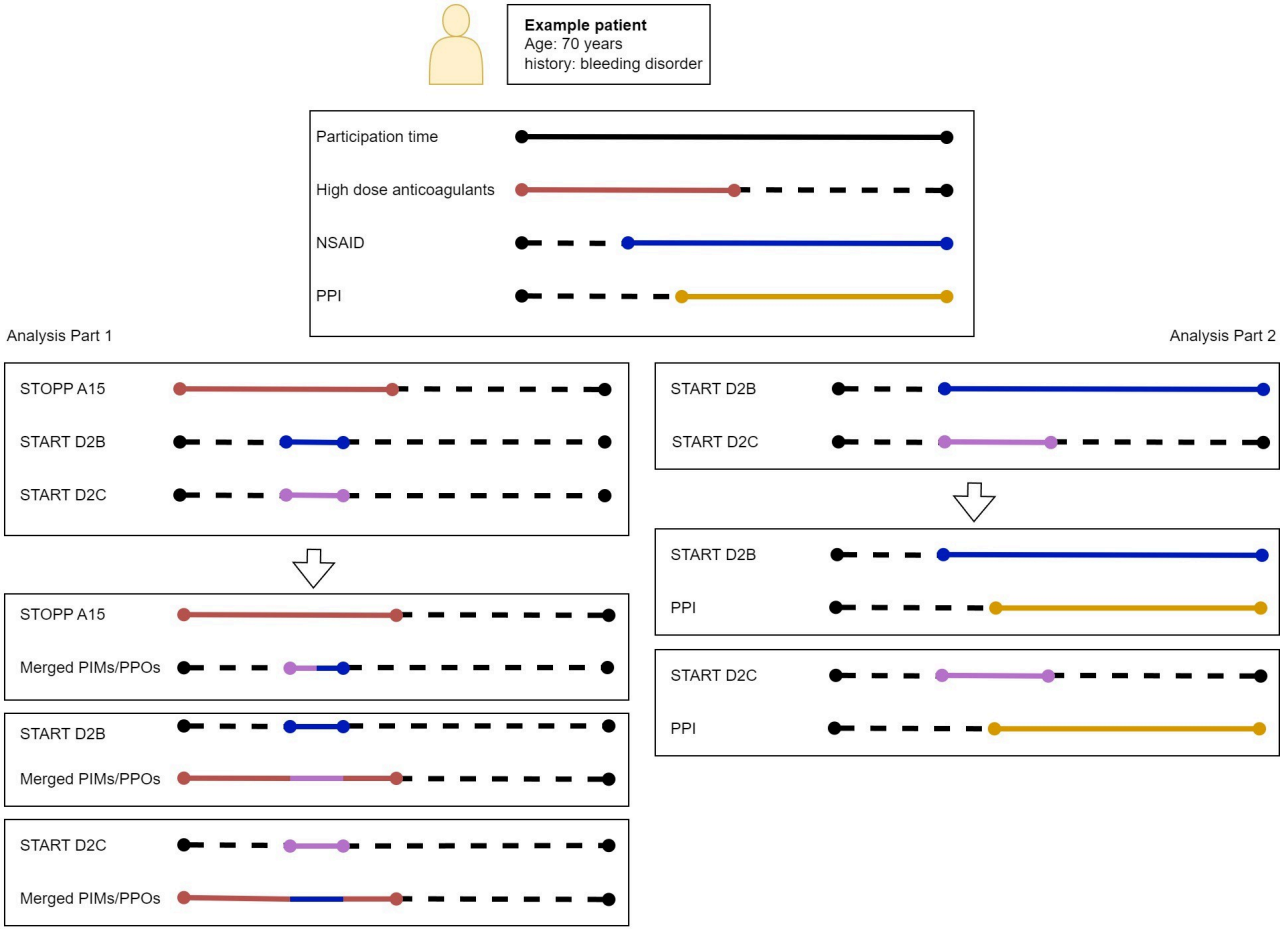
Examples pertaining to part 1 and part 2 of the analysis. The aim of part1 is to assess the association between (i) nonadherence to the STOPPs and the STARTs and (ii) gastrointestinal bleeding. The aim of part2 is to assess the association between (i) the potentially harmful medications and patient characteristics in each START and (ii) gastrointestinal bleeding. For part 1, each potentially harmful medication combination is registered when no PPI was prescribed concomitantly (because a PPI prescription would comply with the criteria), explaining the short sequences of nonadherence to the STARTs. For part 2, the prescriptions are incorporated as is and compared with the PPI prescriptions. Abbreviations: Non-steroid anti-inflammatory drugs–NSAIDs and Proton pump inhibitor–PPI. A description of each STOPP or START code can be found in Table 1.

The possible association between STOPP or START and gastrointestinal bleeding was analysed using a Cox proportional hazards model. Each STOPP or START description consists of a combination of potentially harmful medication(s) and patient characteristics, and each of the STARTs contains an additional condition of gastrointestinal protection in the form of a PPI. Our goal was twofold. First, we want to assess the association between nonadherence to the STOPPs and the STARTs, on the one hand, and gastrointestinal bleeding on the other hand (we refer to this as part 1). Second, we want to assess the association between the potentially harmful medications and patient characteristics in each START, on the one hand, and gastrointestinal bleeding on the other hand (we refer to this as part 2). An example of both parts is shown in Fig 3 for a 70 years old patient with history of bleeding disorder.

For part 1, all cases where PPI was prescribed simultaneously with the medications from the START criteria, this time was labelled as non-exposure. The analysis was performed for all PIMs and PPOs combined, the PIMs combined, the PPOs combined and for each of the nine individual criteria separately. For analysis of combined outcomes, we used only age and sex for adjustment (hereafter AS-adjustment). The individual PIMs and PPOs were analysed in two different models: an AS-adjustment model and a model in which the remaining PIMs and PPOs were included as confounders, after being merged if they overlapped. They were merged using the earliest start date and latest end date (as seen in Figs 2 and 3). For part 2, we included PPI (in accordance with the START criteria) as a binary covariate in order to assess harm due to exposure to possible harmful medication(s) with and without PPI. First, the possible harmful medication(s) and patient characteristics of each START were assessed using a multivariable model. Then, we used an interaction model to analyse the interaction between the potentially harmful medication(s) and PPI. Age and sex were considered as possible confounders in all models. For other possible confounders such as alcohol abuse and smoking, no sufficient data was available. Table 3 displays the statistical formulas for all models.

**Table 3:** The statistical formulas for the models in part 1 and part 2. Age and sex are considered as confounders in all models.

	Model	Statistical formula
Part 1	Multivariable model	$H(t)=h_{0}(t)\times exp(b_{1}\times PIP+b_{2}\times age+b_{3}\times sex)$
	Adjusted model	$H(t)=h_{0}(t)\times exp(b_{1}\times PIP+b_{2}\times PIP\_other+b_{3}\times age+b_{4}\times sex)$
Part 2	Multivariable model	$H(t)=h_{0}(t)\times exp(b_{1}\times meds+b_{2}\times age+b_{3}\times sex)$
	Interaction model	$H(t)=h_{0}(t)\times exp(b_{1}\times PIP+b_{2}\times PPI+b_{3}\times age+b_{4}\times sex+b_{5}\times meds\times PPI)$

Abbreviations: Potential inappropriate prescribing (PIM or PPO)—PIP, Merge of remaining PIMs and PPOs–PIP_other, medication and patient characteristics combination in the STARTs–meds, Proton pump inhibitor–PPI.

**Fig 4:**
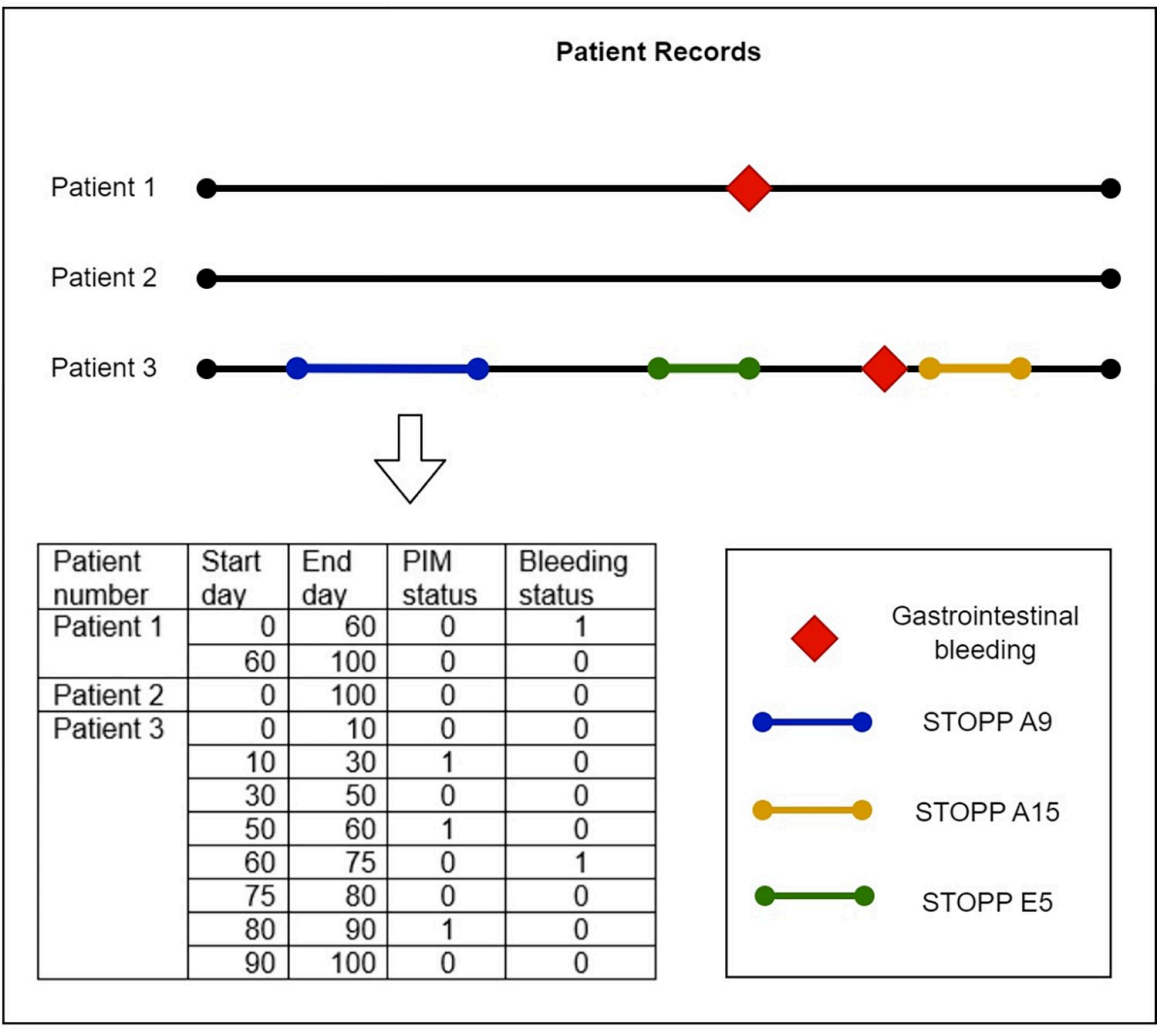
Splitting participants’ time in multiple sequences. Each red diamond indicates a gastrointestinal bleeding, each of the coloured lines indicate (nonadherence to) STOPPs or STARTs (PIM status). The day of each event is given below the patients’ timelines. All participants which did not undergo a PIM or PPO during their time in the study, would have one- or two-time intervals: One spanning from their entry to their exit date (patient 2), or two if they had a gastrointestinal bleeding, which would split their time at the day of the event (patient 1). Patients having one or more PIMs or PPOs could theoretically have up to 2921 sequences, starting a new sequence each time they started or stopped using medication (patient 3).

A participant’s exposure to different medications, PIM or PPO status varies during their time in the study. Therefore, the PIMs and PPOs are time dependent variables, only present when a certain combination of medications is prescribed or omitted. To account for this variation, we performed Cox regression with a time-dependent covariate to capture the PIM and PPO status over time. Specifically, we divided each participant’s time into sequences labelled for exposure to a certain PIM, PPO or no exposure [21]. An example can be found in Fig 4. The Cox model clustered all sequences belonging to the same participant. This adjusts for the clustering effect within the patient. This is also needed because the number of sequences in our control group are lower than in the group having one or more PIMs or PPOs. As a sensitivity analysis, we also analyzed the subpopulation that had experienced bleeding, albeit a small sample. Specifically, we assessed the hazard ratio of PIMs in two scenarios: (i) Any START was considered a PIM; and (ii) only START D3B and START D3C were considered, separately, as a PIM. The choice of these two criteria is based on their emergence as strong association with bleeding in the main analysis.

P-values ≤ 0.05 were considered significant. R Studio version 1.1.463 and R version 3.5.2 were used to perform the analyses, using the packages readr, dplyr, gdata, MASS and survival. All used R-scripts are available from the authors upon request.

## 3. Results

### 3.1 Participants

26,576 participants were included based on age, for the period of time they were registered at the GP practice. The median age was 72 years (IQR 65–82 years). Of the participants, 59% were female. 23,383 (88%) participants did not have any PIM or PPO during the investigated years. A total of 3,193 participants (12%) had one or more PIMs or PPOs, with each PIM or PPO registered as a record. An overview is shown in Table 4.

**Table 4:** Descriptive statistics. The median age (with interquartile range (IQR)), gender distribution, and for the different groups of prescriptions the number of records, number of patients and mean, median and interquartile range duration.

		Part 1	Part 2	
Age		72 years (IQR 65–82)		
Sex		59% ♀		
Prescriptions		PIP	Medication	PPI
**Number of records**		19070	19347	22572
**Patients (n)**		3193	3536	5219
**Percentage patients**		12%	13%	20%
**Duration**	mean	85 days	92 days	123 days
	median	30 days	30 days	89 days
	IQR	12–90	12–90	30–157

Abbreviations: Potential inappropriate prescribing–PIP, Proton pump inhibitor–PPI, Interquartile range–IQR.

### 3.2 Gastrointestinal bleedings

We found 146 gastrointestinal bleedings, which occurred in 143 participants; three patients suffered from two gastrointestinal bleedings during the study. 127 bleedings were identified by specific ICPC codes: we detected 19 cases of hematemesis (ICPC code D14), 36 cases of melena (ICPC code D15), 33 cases of duodenal ulcer (ICPC code D85) and 39 cases of other stomach ulcers (ICPC code D86). The remaining 19 cases were identified by the free text search and inspection, connected to general, non-specific ICPC codes.

### 3.3 Part 1: Association between PIM/PPO and gastrointestinal bleeding

The Cox proportional hazards model for the association between nonadherence to all selected STOPP and START criteria and gastrointestinal bleeding showed a hazard ratio (HR) of 5.45 (CI 3.62–8.21, p-value <0.001). No gastrointestinal bleeding occurred during any of the three PIMs. All results from both the multivariable model and the adjusted model can be found in Table 5, omitting the PIMs due to the absence of gastrointestinal bleedings resulting in no significant results. In the sensitivity analysis in which we analysed the PIMs in the subpopulation that had experienced bleeding we found out that the age and sex adjusted HR in the first scenario in which any START was considered a PIM was 1.34 (CI: 0.98–1.83, p-value = 0.065). In the second scenario the HR of START D3B was 2.11 (CI 1.39–3.20, p-value < 0.001) and of START D3C 1.81 (CI 1.33–2.45, p-value <0.001). Additional results can be found in the appendix.

**Table 5:** For each PIM, PPO or group, the following is provided: The number of occurrences, Hazard Ratio (HR), Confidence interval (95% CI) and p-value. Results are presented for both the basic multivariable model, containing only the PIM or PPO as independent variable (supplemented with age and sex), and the adjusted model, which contains all confounders.

PIM/PPO code	number	Multivariable model		Adjusted model	
		HR (95% CI)	p-value	HR (95%CI)	p-value
START D2A	293	7.62 (1.09–53.54)	0.041*	2.15 (0.30–15.32)	0.445
START D2B	8142	2.92 (1.53–5.59)	0.012*	1.06 (0.49–2.33)	0.878
START D2C	2105	3.23 (1.02–10.22)	0.046*	0.69 (0.21–2.30)	0.547
START D3A	565	2.90 (0.40–20.85)	0.291	1.08 (0.15–7.81)	0.937
START D3B	2968	10.96 (5.78–20.77)	<0.001*	4.72 (2.34–9.48)	<0.001*
START D3C	4763	9.75 (5.79–16.41)	<0.001*	6.96 (3.87–12.53)	<0.001*
STARTs combined	18836	5.53 (3.67–8.32)	<0.001*	-	-
All combined	19070	5.45 (3.62–8.21)	<0.001*	-	-

Significant p-values have been marked with a star (*). Abbreviations: Potentially inappropriate medications–PIM and Potential prescribing omission–PPO. A description of each STOPP or START code can be found in Table 1.

### 3.4 Part 2: Association between potentially harmful medication (PPOs) and gastrointestinal bleeding

The Cox proportional hazards model for the association between exposure to potentially harmful medication(s) and gastrointestinal bleeding showed a hazard ratio of 5.48 (CI 3.64–8.25, p-value <0.001). When a PPI was added during exposure to these medications (in accordance with all six PPO criteria, as seen in Table 3: Part 2, interaction model), this resulted in an HR of 0.70 (CI 0.18–2.66, interaction coefficient -2.24, p-value < 0.001). Exposure to only a PPI increases the hazard of gastrointestinal bleedings (HR 10.84, CI 7.58–15.50, p-value <0.001). Table 6 displays the results, omitting interaction results for PPOs during which too few gastrointestinal bleedings occurred resulting, again, in no significant results. The Appendix contains tables with all outcomes for part 2.

**Table 6:** For each PPO or group the following is provided: START code, number of occurrences, Hazard Ratio (HR), Confidence interval (95% CI), p-value and coefficient. The interaction models are split in results for exposure to PPI or no exposure to PPI.

PPO code	number	Multivariable model		Interaction model			
		HR (95%CI)	p-value	PPI HR	no PPI HR (95%CI)	coeff	p-value
START D2A	307	7.16 (1.02–50.32)	0.048*	-	18.57 (2.58–133.8)	-16.30	<0.001*
START D2B	8458	3.61 (2.03–6.43)	<0.001*	0.56 (0.18–1.76)	4.64 (2.24–9.63)	-2.11	<0.001*
START D2C	2187	3.03 (0.96–9.56)	0.059	0.60 (0.30–1.20)	2.61 (0.36–18.70)	-1.47	0.238
START D3A	540	2.76 (0.38–19.80)	0.313	-	8.40 (1.15–61.40)	-17.46	<0.001*
START D3B	2996	13.09 (7.41–23.14)	<0.001*	2.29 (1.07–4.89)	13.41 (5.86–30.69)	-1.77	0.002*
START D3C	4859	10.53 (6.45–17.19)	<0.001*	1.36 (0.55–3.36)	12.43 (6.93–22.30)	-2.21	<0.001*
All STARTs	19347	5.48 (3.64–8.25)	<0.001*	0.70 (0.18–2.66)	6.63 (3.97–11.06)	-2.24	<0.001*

We provided the Hazard Ratio (HR) and Interaction coefficient (coeff) with p-value of each interaction model. Significant p-values have been marked with a star (*). Abbreviations: Potential Prescribing omission–PPO, Proton pump inhibitor–PPI, coefficients–coeff. A description of each STOPP or START code can be found in Table 1.

## 4. Discussion

### 4.1 Main findings

This study demonstrates the hazard of gastrointestinal bleedings in older patients increases when one does not adhere to the specific STOPPs and STARTs (HR 5.45, CI 3.62–8.21, p-value <0.001). Regarding the START criteria, exposure to the potentially harmful medications increases the hazard of gastrointestinal bleedings (HR 5.48, CI 3.64–8.25, p-value <0.001). The hazard of gastrointestinal bleedings for each separate criterion showed much variability (HR between 0.69 and 6.96), of which just START D3B (HR 4.72, CI 2.34–9.48, p-value <0.001) and START D3C (6.96, CI 3.87–12.53, p-value <0.001) were significantly associated with an increased hazard of gastrointestinal bleeding.

### 4.2 Discussion

Variability exists between the individual criteria, both in number of records and overall effect. No gastrointestinal bleedings occurred during any of the STOPPs, which implies a hazard ratio close to zero. However, the number of records for each of the STOPPs was low (between 10 and 142 records) compared to the STARTs (between 293 and 8142 records). For two STARTs for which the combination of potentially harmful medication(s), patient characteristics and gastrointestinal bleeding were so scarce, results are difficult to interpret. When focussing on the potentially harmful medication combinations of the other four STARTs, START D2B and START D2C showed a possible protective effect of PPIs on the gastrointestinal tract, with a combination of a decreased HR when a PPI was prescribed and an increased HR when a PPI was omitted, although not all results were significant. The last two START combinations of potentially harmful medication(s) and patient characteristics, START D3B and START D3C, showed both an increased HR for PPI prescription and PPI omission. Although their HRs for PPI omittance were higher than any other, it implies the medications prescribed inflict more damage to the gastrointestinal tract than a concomitant PPI can avert. Five out of six STARTs showed a significant association with gastrointestinal bleeding in the multivariable models, significant associations in the adjusted models were sparse. Only two out of nine criteria showed significantly increased hazard ratios in the adjusted models. Both of these criteria are based on a prescription for anticoagulants and advanced age (>70 or >80 years old). Despite the small sample of the subpopulation that experienced bleeding, our sensitivity analysis in this group corroborates our main analysis by showing elevated hazard ratios of the PIMs (taken as a whole) and especially when taken separately for START D3B and START D3C".

Surprisingly, exposure to PPI resulted in a significant association with gastrointestinal bleedings. We expect a group of patients registered as using only a PPI, to take both NSAID (sold over the counter (OTC)) and PPI. 13% of patients at risk for ADRs use these NSAIDs without a prescription [22]. Several other medications named in the STOPPs and STARTs are available as OTC medication. Although OTC medications are not recorded in the EHR, they might be discussed during consultations. Risk factors such as alcohol consumption, Helicobacter Pylori infection, smoking, unmarried status and cardiovascular disease [23–25] could not be included in our study. Other studies have shown that for a substantial part of the older patient population a PPI is prescribed and this number is rising [26–28]. It is possible GPs weigh in risk factors and use of OTC medications and are adapting their PPI prescribing habits accordingly. Based on our results, these patients might have an even greater risk of gastrointestinal bleedings if GPs would omit prescribing PPIs to these patients.

### 4.3 Strengths and limitations

This is one of the first studies assessing the preventive capacity of the STOPPs and STARTs on ADRs, and the first on gastrointestinal bleeding, in large dataset. This multicentre study, using data from eight consecutive years, performed multiple model analysis considering time dependent variables. Although a large dataset was available, the number of gastrointestinal bleedings was limited, resulting in few or no bleedings during some of the individual STOPPs and STARTs. Furthermore, we expect physicians to prescribe PPI for patients sharing information on risk factors or use of OTC medications, not registered in the dataset, and therefore not included in our models. Other limitations are the retrospective design of the study and omission of some risk factors as confounders due to their absence in the dataset used.

### 4.4 Literature

We used and adapted the specified PIMs and PPOs from a previous study [14]. Our results underline their claim to the importance of implementing the STOPP and START guideline in practice. Most studies report on prevalence of nonadherence to the STOPP and START guideline, without taking prevalence of resulting ADRs into account. This study shows a common PIP does not always lead to an increased hazard of the ADR it is designed to prevent. Furthermore, a multicenter study into common ADRs as a result of PIP found that gastrointestinal bleedings are amongst the most common preventable ADRs [29]. However, when focussing on the selected STOPPs and STARTs from our study, they found START D2A, START D3A, STOPP A15 and STOPP E5 were the most frequent PIPs. This conflicts with our results, since these STOPPs and STARTs were amongst the least frequent PIPs. However, the interaction coefficient for START D2A and D3A and PPI was high, suggesting a significant protective effect of PPI for these patients. Other studies show a difference in PIP prevalence between settings [9,30,31]. Additionally, sharing information on medication and admissions or treatments between hospitals and GPs could be improved [32,33]. As the earlier study focussed on 301 admitted hospital patients, and ours included almost 27,000 participants from the general population, the difference in population and setting might explain the contrasting results [29]. As our study only includes GP data, we might have missed gastrointestinal bleedings treated in the hospital, which were not correctly recorded at the GP.

### 4.5 Implications

With 87 individual criteria, the comprehensiveness of the STOPP and START guideline could be a barrier for implementation [34]. This study shows not all STOPPs and STARTs are equally effective or occurrent, and therefore a selection of criteria could be implemented in supporting tools to prevent alert fatigue [35]. The Dutch multidisciplinary guideline in which the adapted STOPPs and STARTs are published also contains recommendation for medication review boards. Since NSAIDs, ASA, ECC and PPI are all commonly prescribed, we expect the review boards to regularly encounter the selected STOPPs and STARTs from this study. By assessing the effectiveness of the criteria, and providing context to the interacting effects, we hope to alleviate part of their reviewing tasks.

### 4.6 Future work

Future research into the effectivity of the criteria for other ADRs, using the same methods, should be performed. Especially since some of the criteria with an increased prevalence seem to be superfluous, like START D2C. The association with gastrointestinal bleeding for five out of nine criteria was difficult to assess due to a low number of records. We thus suggest using a larger dataset, possibly combining different regions for generalizability. This data would have to contain information on risk factors described above, notes on OTC medications discussed during consultations, data on hospital admissions and pharmacy records to included information on collection of medication [36]. Strategies to improve adherence to the STOPP and START guideline could include the development and employment of decision support systems to advise physicians on their prescribing habits.

## 5. Conclusion

This study shows the harmful effects of certain medications on the gastrointestinal tract. Given this warning, physicians should be cautious when prescribing these medications to older patients. Furthermore, our results emphasize the importance of the STOPP and START guideline in preventing possible inappropriate prescribing to reduce the risk of gastrointestinal bleedings in older people. This may also hold for other ADRs. The method we used can be applied in future research, focussing on other STOPPs and STARTs.

## Supporting information

S1 Table Cox model outcomes for each PIM or PPO. Notice the extreme low outcomes for all PIMs due to absence of bleedings. The number of records and the number of unique patients these records belong to are shared for each PIM or PPO. Abbreviations: Hazard Ratio–HR, Confidence Interval–CI, Acetylsalicylic acid–ASA, Effervescent carbasalate calcium–ECC, Non-Steroidal Anti-Inflammatory Drug–NSAID, Proton Pump Inhibitor–PPI, and Selective Serotonin Reuptake Inhibitors—SSRI. Significant p-values have been marked with a star (*).
(DOCX)

S2 Table PPO’s and corresponding ratio’s. For each PPO or group the number of occurrences, Hazard Ratio (HR), Confidence interval (CI) and p-value are given. Results are presented for both the HR of potentially inappropriate prescribing (PIP), and the interaction model, which contains the interaction between PIP and PPI. The resulting Hazard Ratios (HR), 95% confidence intervals (95% CI) and p-values are given. Significant p-values have been marked with a star (*).
(DOCX)

S3 Table The coefficients for all variables of each interaction model with the corresponding p-value. Significant p-values have been marked with a star (*).
(DOCX)
