# Supplementary material for: Time-dependent association between STOPP and START criteria and gastrointestinal bleeding in older patients using routinely collected primary care data
Source: PLoS One. 2023 Dec 7;18(12):e0292161. doi: 10.1371/journal.pone.0292161 (PMC10703206; doi:10.1371/journal.pone.0292161)
Supplement: S1 Table — Notice the extreme low outcomes for all PIMs due to absence of bleedings. The number of records and the number of unique patients these records belong to are shared for each PIM or PPO. Abbreviations: Hazard Ratio–HR, Confidence Interval–CI, Acetylsalicylic acid–ASA, Effervescent carbasalate calcium–ECC, Non-Steroidal Anti-Inflammatory Drug–NSAID, Proton Pump Inhibitor–PPI, and Selective Serotonin Reuptake Inhibitors—SSRI. Significant p-values have been marked with a star (*). (DOCX) [file pone.0292161.s001.docx]

*Table 7: Cox model outcomes for each PIM or PPO. Notice the extreme low outcomes for all PIMs due to absence of bleedings. The number of records and the number of unique patients these records belong to are shared for each PIM or PPO. Abbreviations: Hazard Ratio – HR, Confidence Interval – CI, Acetylsalicylic acid – ASA, Effervescent carbasalate calcium – ECC, Non-Steroidal Anti-Inflammatory Drug – NSAID, Proton Pump Inhibitor – PPI, and Selective Serotonin Reuptake Inhibitors - SSRI. Significant p-values have been marked with a star (*).*

| **ASA and dose >160mg/day or ECC dose > 200mg/day** | | | | | | |
| --- | --- | --- | --- | --- | --- | --- |
| PIM code | HR (95% CI) – multivariable | p-value | HR (95% CI) – adjusted | p-value | Records | Patients |
| STOPP A9 | 1.61e-5 (1.08e-5-2.40e-5) | <0.001* | 5.48e-6 (3.07e-6-9.76e-6) | 0.001* | 82 | 53 |
| **platelet aggregation inhibitors or oral anticoagulants and concurrent bleeding disorder** | | | | | | |
| PIM code | HR (95% CI) – multivariable | p-value | HR (95% CI) – adjusted | p-value | Records | Patients |
| STOPP A15 | 1.21e-4 (2.95e-5-4.95e-4) | <0.001* | 2.48e-5(4.33e-6-1.42e-4) | <0.001* | 10 | 3 |
| **NSAID during >3 months for gout without contraindication or proven ineffectiveness for allopurinol** | | | | | | |
| PIM code | HR (95% CI) – multivariable | p-value | HR (95% CI) – adjusted | p-value | Records | Patients |
| STOPP E5 | 5.85e-6 (4.00e-6-8.54e-6) | <0.001* | 1.22e-6(7.50e-7-1.99e-6) | <0.001* | 142 | 57 |
| **NSAID and history of peptic ulcer disease or complication from peptic ulcer disease without PPI** | | | | | | |
| PPO code | HR (95% CI) – multivariable | p-value | HR (95% CI) – adjusted | p-value | Records | Patients |
| START D2A | 7.62 (1.09 – 53.54) | 0.0411* | 2.15 (0.30 – 15.32) | 0.445 | 293 | 86 |
| **patients aged >70 years using NSAID without PPI** | | | | | | |
| PPO code | HR (95% CI) – multivariable | p-value | HR (95% CI) – adjusted | p-value | Records | Patients |
| START D2B | 2.92 (1.53 – 5.59) | 0.0012* | 1.06 (0.49 – 2.33) | 0.878 | 8142 | 2171 |
| **NSAID in patients 60-70 years and oral anticoagulants or oral corticosteroids or SSRI or ASA or ECC without PPI** | | | | | | |
| PPO code | HR (95% CI) – multivariable | p-value | HR (95% CI) – adjusted | p-value | Records | Patients |
| START D2C | 3.23 (1.02 – 10.22) | 0.0459* | 0.69 (0.21 – 2.30) | 0.547 | 2105 | 723 |
| **ASA or ECC and >60 years and history of peptic ulcer without PPI** | | | | | | |
| PPO code | HR (95% CI) – multivariable | p-value | HR (95% CI) – adjusted | p-value | Records | Patients |
| STOPP D3A | 2.90 (0.40 – 20.85) | 0.291 | 1.08 (0.15 – 7.81) | 0.937 | 565 | 117 |
| **ASA or ECC and >70 years and oral anticoagulants or oral corticosteroids or SSRI without PPI** | | | | | | |
| PPO code | HR (95% CI) – multivariable | p-value | HR (95% CI) – adjusted | p-value | Records | Patients |
| START D3B | 10.96 (5.78 – 20.77) | <0.001* | 4.72 (2.34 – 9.48) | <0.001* | 2968 | 834 |
| **ASA or ECC and >80 years without PPI** | | | | | | |
| PPO code | HR (95% CI) – multivariable | p-value | HR (95% CI) – adjusted | p-value | Records | Patients |
| START D3C | 9.75 (5.79 – 16.41) | <0.001* | 6.96 (3.87 – 12.53) | <0.001* | 4763 | 1139 |
