# Supplementary material for: Time-dependent association between STOPP and START criteria and gastrointestinal bleeding in older patients using routinely collected primary care data
Source: PLoS One. 2023 Dec 7;18(12):e0292161. doi: 10.1371/journal.pone.0292161 (PMC10703206; doi:10.1371/journal.pone.0292161)
Supplement: S2 Table — For each PPO or group the number of occurrences, Hazard Ratio (HR), Confidence interval (CI) and p-value are given. Results are presented for both the HR of potentially inappropriate prescribing (PIP), and the interaction model, which contains the interaction between PIP and PPI. The resulting Hazard Ratios (HR), 95% confidence intervals (95% CI) and p-values are given. Significant p-values have been marked with a star (*). (DOCX) [file pone.0292161.s002.docx]

*Table 8: PPO’s and corresponding ratio’s. For each PPO or group the number of occurrences, Hazard Ratio (HR), Confidence interval (CI) and p-value are given. Results are presented for both the HR of potentially inappropriate prescribing (PIP), and the interaction model, which contains the interaction between PIP and PPI. The resulting Hazard Ratios (HR), 95% confidence intervals (95% CI) and p-values are given. Significant p-values have been marked with a star (*).*

| **PPO code** | **number** | **Patients** | **Multivariable model** | | **Interaction model** | | | |
| --- | --- | --- | --- | --- | --- | --- | --- | --- |
|  |  |  | **HR (95%CI)** | **p-value** | **PPI**  **HR** | **no PPI**  **HR (95%CI)** | **coeff** | **p-value** |
| START D2A | 307 | 98 | 7.16  (1.02 – 50.32) | 0.048* | 1.64e-6  (2.13e-6-1.27e-6) | 18.57  (2.58 – 133.8) | -16.30 | <0.001* |
| START D2B | 8458 | 2452 | 3.61  (2.03 – 6.43) | <0.001* | 0.56  (0.18 - 1.76) | 4.64  (2.24 – 9.63) | -2.11 | <0.001* |
| START D2C | 2187 | 800 | 3.03  (0.96 – 9.56) | 0.059 | 0.60  (0.30 - 1.20) | 2.61  (0.36 – 18.70) | -1.47 | 0.238 |
| START D3A | 540 | 131 | 2.76  (0.38 – 19.80) | 0.313 | 2.19e-7  (1.53e-7–3.12e-7) | 8.40  (1.15 – 61.40) | -17.46 | <0.001* |
| START D3B | 2996 | 913 | 13.09  (7.41 – 23.14) | <0.001* | 2.29  (1.07 - 4.89) | 13.41  (5.86 – 30.69) | -1.77 | 0.002* |
| START D3C | 4859 | 1313 | 10.53  (6.45 – 17.19) | <0.001* | 1.36  (0.55 - 3.36) | 12.43  (6.93 – 22.30) | -2.21 | <0.001* |
| All STARTs | 19029 | 3536 | 5.48  (3.64 – 8.25) | <0.001* | 0.70  (0.18 - 2.66) | 6.63  (3.97 – 11.06) | -2.24 | <0.001* |
