# Supplementary material for: Time-dependent association between STOPP and START criteria and gastrointestinal bleeding in older patients using routinely collected primary care data
Source: PLoS One. 2023 Dec 7;18(12):e0292161. doi: 10.1371/journal.pone.0292161 (PMC10703206; doi:10.1371/journal.pone.0292161)
Supplement: S3 Table — Significant p-values have been marked with a star (*). (DOCX) [file pone.0292161.s003.docx]

**Table 9: The coefficients for all variables of each interaction model with the corresponding p-value.** Significant p-values have been marked with a star (*).

|  | **START D2A** | | **START D2B** | | **START D2C** | |
| --- | --- | --- | --- | --- | --- | --- |
|  | **coefficient** | **p-value** | **coefficient** | **p-value** | **coefficient** | **p-value** |
| PPO | 2.40 | <0.001* | 2.53 | <0.001* | 2.42 | <0.001* |
| PPI | 2.92 | 0.004 | 1.53 | <0.001* | 0.96 | 0.339 |
| Age | 0.01 | 0.440 | 0.01 | 0.365 | 0.01 | 0.431 |
| Sex | -0.19 | 0.235 | -0.20 | 0.239 | -0.19 | 0.245 |
| Interaction | -16.30 | <0.001* | -2.11 | <0.001* | -1.47 | 0.238 |
|  | **START D3A** | | **START D3B** | | **START D3C** | |
|  | **coefficient** | **p-value** | **coefficient** | **p-value** | **coefficient** | **p-value** |
| PPO | 2.42 | <0.001* | 2.32 | <0.001* | 2.45 | <0.001* |
| PPI | 2.13 | 0.036 | 2.60 | <0.001* | 2.52 | <0.001* |
| Age | 0.01 | 0.428 | 0.01 | 0.601 | -0.01 | 0.577 |
| Sex | -0.20 | 0.233 | -0.19 | 0.254 | -0.19 | 0.250 |
| Interaction | -17.46 | <0.001* | -1.77 | 0.002 | -2.21 | <0.001* |
